# Hearing Preservation in Cochlear Implant Surgery

**DOI:** 10.1155/2014/468515

**Published:** 2014-09-03

**Authors:** Priscila Carvalho Miranda, André Luiz Lopes Sampaio, Rafaela Aquino Fernandes Lopes, Alessandra Ramos Venosa, Carlos Augusto Costa Pires de Oliveira

**Affiliations:** ^1^Brasília University Hospital, Hospital Universitário de Brasília-HUB, SGAN 605, Avenida L2 Norte, 70830-200 Brasília, DF, Brazil; ^2^Universidade de Brasília (UnB), Campus Universitário Darcy Ribeiro, 70910-900 Brasília, DF, Brazil

## Abstract

In the past, it was thought that hearing loss patients with residual low-frequency hearing would not be good candidates for cochlear implantation since insertion was expected to induce inner ear trauma. Recent advances in electrode design and surgical techniques have made the preservation of residual low-frequency hearing achievable and desirable. The importance of preserving residual low-frequency hearing cannot be underestimated in light of the added benefit of hearing in noisy atmospheres and in music quality. The concept of electrical and acoustic stimulation involves electrically stimulating the nonfunctional, high-frequency region of the cochlea with a cochlear implant and applying a hearing aid in the low-frequency range. The principle of preserving low-frequency hearing by a “soft surgery” cochlear implantation could also be useful to the population of children who might profit from regenerative hair cell therapy in the future. Main aspects of low-frequency hearing preservation surgery are discussed in this review: its brief history, electrode design, principles and advantages of electric-acoustic stimulation, surgical technique, and further implications of this new treatment possibility for hearing impaired patients.

## 1. Introduction

In the past three decades, cochlear implantation has evolved from an experimental procedure to represent the standard of care for deaf patients. Advances in processing strategies, implant design, and patient selection criteria have significantly improved implant users' performance. Nowadays, the current frontiers in implantation involve strategies to preserve residual acoustic hearing and the development of algorithms to combine electrical and acoustic hearing. Moreover, it is important to keep in mind that, by preserving apical organ of Corti structures, it is possible to take advantage of new technologies that may lead to regeneration of the inner ear in the future [1].

Cochlear implantation with a standard-length electrode has been a reality in treating patients who have profound deafness. However, the loss of residual acoustic hearing following cochlear implantation is an important clinical consideration when determining the most appropriate options for patients with severe hearing losses [2]. There are some patients with substantial low-frequency acoustic hearing up to 1500 Hz and severe to profound high-frequency hearing loss that do poorly with bilateral amplification who have not been considered as candidates for implantation using standard criteria [3].

High frequencies report information about vocal vibration (like the ability to distinguish between “s” and “z”), whereas lower frequencies apprise information regarding the vocal formants and spectral patterns (such as the difference between “b” and “g”) [4]. Patients with high-frequency losses are able to distinguish loudness and speech pattern (due to their low-frequency acoustic hearing), but they cannot interpret spectral patterns well (which erodes their capacity to distinguish between the different consonant sounds and thus their word discrimination scores) [5].

The loss of low-frequency hearing during cochlear implantation is the result of the technique used to create the cochleostomy and its size combined with the characteristics of the electrode design (diameter, stiffness, and length) since it may induce substantial damage to the basilar membrane and cochlear hair cells as it advances into the scala tympani [1, 2].

## 2. Electric-Acoustic Stimulation (EAS)

### 2.1. Shortened Electrodes

It is known that cochlear implantation with a standard-length electrode and standard surgical technique in patients with some residual hearing results in complete loss of the remaining acoustic hearing [6, 7]. Nevertheless, since Hodges et al. [8] presented a series of patients who had undergone implantation and had preserved residual hearing, many studies have demonstrated the ability to retain residual low-frequency hearing in standard-length electrode implantation [9, 10].

The idea of acoustic plus electric hearing means a cochlear implant aided by an ipsilateral hearing aid, to benefit from the residual low-frequency hearing of an individual. This idea of electric-acoustic stimulation (EAS) first emerged from the work of two independent groups, one from Iowa, USA, and the other from Frankfurt, Germany. In 1995, the University of Iowa Cochlear Implant research team along with the Cochlear Corporation (Lane Cove, Australia) developed a shortened electrode array called the Hybrid S. It was designed to be inserted only into the lower basal turn of the cochlea and thus stimulate the missing areas of high frequency for those specific patients with preserved low-frequency thresholds [2]. The Hybrid S electrode has a smaller diameter than the standard electrode (0.2 mm × 0.4 mm) and initially, it was 6 mm in length and it contained 6 electrodes. Because some patients reported a very high-pitched sound [11], it was then lengthened to 10 mm, still with 6 electrodes. The ideal insertion depth is at approximately 195° of the basal turn of the cochlea [12].

In 2003, Gantz and Turner [11] reported the first 6 patients implanted with the Hybrid S electrode, three of which received the 6 mm electrode and the other three received the 10 mm electrode. Patients who received the 6 mm electrode improved their consonant recognition scores by 10%, whereas those who received the 10 mm electrode improved by 40%. Also, patients who received the 10 mm electrode did better in the combined mode (cochlear implant + bilateral hearing aids) than those who received the 6 mm electrode [13]. A larger multicenter phase 1 FDA trial was conducted for the Hybrid S 10 mm electrode with 87 patients from 13 centers, and preliminary data was published in 2009 [14]. Two patients lost all residual hearing within 1 month of implantation (initial hearing preservation rate (IHPR) of 98%). Between 3 and 24 months after activation, 6 more patients lost residual hearing (IHPR of 91%). Over time, 30% of the patients had a low-frequency threshold drop of more than 30 decibels (dB). A duration of deafness of over 40 years and low preoperative consonant-nucleus-consonant (CNC) word scores were found to have a negative impact on functional outcomes [14].

Another short electrode named Hybrid L24 has been developed in conjunction with the Cochlear Corporation. It contains 22 electrodes that are 16 mm long and its optimal insertion is 250° of the basal turn of the cochlea. It would still preserve the residual hearing from the apical portions of the cochlea and if the low-frequency hearing is lost, it can be used as a traditional electric device since it has 22 electrodes, similar to a standard one. The FDA trial for the Hybrid L has not been published yet, but the preliminary results from the European clinical trial [15] demonstrated the ability to preserve residual hearing. Thirty-two patients were enrolled, 24 of whom were hybrid candidates and 8 long-electrode candidates. In 96% of the subjects, hearing was preserved within 30 dB of preoperative thresholds and in 68% within 15 dB. These results were stable over time and there was a significant improvement in word scores between the 6-month and 12-month marks, demonstrating, as in the Hybrid S trial, that there is a learning period for patients with short electrodes [16].

The Med-El Corporation has also developed a shortened electrode called M, which is 22 mm long with an ideal insertion of 360° from the basal turn of the cochlea. It has a very flexible tip and a significantly reduced diameter in the distal portion. This Flex^EAS^ electrode can be used for both round window insertion and cochleostomy techniques [17]. Much of the data regarding Flex^EAS^ involves mixed cohorts of patients. Gstoettner et al. in 2008 [18] reported a series of 18 patients implanted with the M electrode. Twelve of the 18 (68%) had low-frequency hearing preservation that could be usefully amplified. Three of the patients had some residual low-frequency hearing but did not find amplification useful. Three of the 18 (16%) lost all residual hearing. Interestingly, the loss of the residual hearing was not immediate, but delayed by 3 to 6 months after hybrid activation.

### 2.2. Standard-Length Electrodes

An alternative strategy for preserving the low-frequency hearing was developed with standard-length electrodes, but limiting the depth of insertion. Kiefer et al. [7] implanted 14 patients with the Med-El Combi40+ electrode, limiting the length of insertion to less than 24 mm (full insertion is 31.5 mm) and using a “soft insertion” technique. In 12 out of the 14 patients (85%), useful low-frequency hearing (less than 20 dB drop in thresholds) was maintained, with 2 patients losing all residual hearing. Using a standard-length electrode and modified surgical techniques, other authors have reported rates from 67% to 89% of hearing preserved within 20 dB of preoperative thresholds [7, 19–22]. Nevertheless, not all of these patients maintain the ability to discriminate. Balkany et al. reported that although patients experienced only a 15 dB drop in the low frequencies, the average acoustic CNC postoperative word score was 0% [9].

### 2.3. Discussion

There is controversy in the literature about the preferred electrode length. There is a higher rate of reduced thresholds and anacusis with the long electrodes. Some authors defend this reason for its use, so if residual hearing is lost, the patient can have the full-length electrode to benefit from its electric-only listening mode, which would not happen with a short electrode. Since the short electrode is 10 mm long, it accesses only the 2800 to 4700 Hz range according to the Greenwood frequency placement map of the basilar membrane. Even though this was thought to be a tonotopic mismatch, which could impair discrimination, Hybrid S electrode users in electric-only mode showed similar performance as long-electrode users on consonant recognition tasks [23]. Improved performance with the Hybrid S electrode appears to require a longer time (over 12 months) than the long-electrode (usually 6 to 12 months to adapt to electric hearing) [16, 23].

One argument in favor of long electrode used to be the likelihood of progressive low-frequency hearing loss. However, Yao et al. [24] demonstrated a loss of only 1.05 dB in low-frequency hearing (up to 2000 Hz) per year in Hybrid S users, regardless of their age. It seems that low-frequency hearing is relatively stable over time. If it can be preserved at the time of implantation, it is likely that the patient will have minimal further hearing loss in the long term [24].

Another concern regarding Hybrid or EAS studies is the progression of hearing loss after activation. At the time of implantation, very few patients lose all their residual low-frequency hearing. In the phase 1 Hybrid S10 trial [14], 2% of the patients lost their residual low-frequency hearing, and within 3 months of activation, 10% of the initial number of patients had a 30 dB drop from their preoperative thresholds up to 500 Hz. The cause of this loss is still unknown; some hypotheses suggest an immune reaction to the electrode, loss of afferent spiral ganglion neuron synapses at the hair cell related to the combination of acoustic amplification and electrical stimulation, or even an initial injury from noise-induced hearing loss [25].

Steroids have been shown to reduce noise-induced cochlear damage and hearing loss and to increase recovery after noise trauma. However, their efficacy has been controversially discussed due to a lack of adequate clinical trials [1]. Also, there is controversy regarding drug application methods, be it systemic or local, either via diffusion from the middle ear space through the round window membrane or by direct instillation into the perilymphatic space. A single-shot intracochlear glucocorticoid application appears to be a promising method for reducing progressive hearing loss caused by electrode insertion trauma due its long-term effects, such as reduction of inflammatory processes [1]. Further in vitro studies with otoprotective drugs believed to bring new perspectives on an improved rate of hearing preservation are promising [1, 26, 27].

## 3. Indication Guidelines for EAS

In the initial studies, the indication was closer to standard cochlear implantation for thresholds above 65 dB in the low frequencies between 125 and 500 Hz [28]. After encouraging results from these studies, the criteria were gradually expanded to normal low-frequency hearing in the frequencies up to 1500 Hz (partial deafness cochlear implantation) [29].

Actual guidelines determine that pure tone audiometry scores for both ears have to be greater than 60 dB between 125 and 500 Hz and below 70 dB at 1500. In addition, monosyllables tested at a 10 dB signal-to-noise ratio (SNR) should not exceed a score of 40% in the best aided condition [1]. Further, these patients must have substantial CNC word scores in the best aided condition, with between 10% and 60% correct in the worse hearing ear and up to 80% correct in the best hearing ear.

## 4. Surgical Technique for Hearing Preservation

Wright and Roland in 2005 [30] first described a “soft surgery” technique, which was later modified by other authors [7, 12, 13]. When developing new strategies, the most import factors that contribute to possible cochlear damage during or after the surgery must be kept in mind [1]:

- mechanical damage during electrode insertion (fractures of the osseous spiral lamina, disruption of the basilar membrane, tearing of the lateral spiral ligament, and leakage of traumatized blood vessels),
- shock waves in the perilymph fluid due to implantation,
- acoustic trauma due to drilling,
- loss of perilymph and disruption of inner ear fluid homeostasis,
- potential bacterial infection,
- secondary intracochlear fibrous tissue formation.

The technique used at the University of Iowa for implantation of the cochlear Hybrid S and L devices is described in this review.

A standard mastoidectomy is performed through a postauricular incision. A portion of the superior margin of the mastoid cortex is left in place and a suture is passed through the cortex to anchor the electrode before placing it in the cochlea. The objective of this suture is to reduce the spring of the electrode and to prevent movement of the electrode during placement in the scala tympani (ST). A bonny well for the internal processor is also created in the same manner used for a standard cochlear receiver/stimulator. A ridge of bone between the mastoid cavity and the well is left so the implant does not later slide. A subperiosteal pocket is created deeply to the temporalis muscle and pericranium. The facial recess is opened widely, and the round window niche is totally exposed by removing the bone overlying and anterior to the facial nerve. With a 1 mm diamond burr, the bony overhang of the round window niche is drilled away to expose the entire round window membrane.

Before creating the cochleostomy, the surgical field must be extensively irrigated and meticulous hemostasis must be done in order to prevent bone chips and blood debris from entering into the cochlea upon opening. The cochleostomy is created in the inferior-posterior quadrant of a box created by drawing a line at the superior margin of the round window and a perpendicular line at the inferior margin of the round window [2]. Entering into the scala tympani in this specific location prevents damage to the basilar membrane and avoids injury to inner ear structures by facilitating insertion of the electrodes in the correct trajectory [3]. Although in the United States a cochleostomy is the preferred strategy for placing the electrode, many European surgeons usually prefer the round window approach [3]. The round window technique may result in a conductive hearing loss [15] and it predisposes to fracturing the osseous spiral lamina, according to some studies [30, 31]. A recent systematic review [32], however, did not show any benefit of one surgical approach over the other regarding the preservation of residual hearing.

The electrode and processor must be seated in position before opening the endosteum of the cochleostomy. Two holes are drilled into the tegmen tympani, which allow a 2-0 or 4-0 nylon suture to be passed through the bone in order to secure the Hybrid S and L electrode array (not the ground electrode). Once the processor and electrode are seated and secured, a temporalis fascia “washer” with a 1.5 mm × 1.5 mm diameter is harvested and flattened in a fascial tissue press. It is then punctured with a straight needle and the electrode is inserted through the fascia up to the Dacron collar in order to seal the scala tympani at the cochleostomy. Opening the endosteum into the scala tympani with a 0.2 mm footplate hook must be the final act of the procedure to reduce the time the cochlea is open. The electrode is advanced slowly into the scala tympani (over 1 to 2 minutes) in order to minimize insertional trauma and to allow perilymph displacement.

There are some intraoperative tests described in order to further improve the safety of hearing preservation surgery, such as the measure of cochlear microphonics [1] and auditory brainstem response (ABR) during electrode insertion [3]. Likewise, Oghalai et al. [33] used auditory steady-state response audiometry to access hearing thresholds during regular cochlear implantation and draw the surgeon's attention to the critical moments of the insertion procedure. The surgeon must pause during the insertion to allow data for these tests to be collected.

The middle ear is not packed with muscle or fascia. The periosteum should then be closed completely over the receiver/stimulator and the electrode. Soft tissues are then closed in the standard way.

Helbig et al. [34] recently reported a series of 3 patients in whom revision surgery for cochlear implantation was required. Reimplantation was possible in these patients who had previously undergone EAS surgery with the preservation of low-frequency hearing and without losing the residual hearing function. Also, a reduced insertion depth at the initial surgery could be followed by deeper insertion into the cochlea without any deterioration of residual hearing [34].

## 5. Benefits of Acoustic and Electric Processing

Current literature has already reported several discrete advantages to both modalities, but the full benefits of an EAS of the ipsilateral cochlea are still under investigation.

### 5.1. Speech Perception with EAS

Patients with preserved low-frequency hearing show significant improvements in their discrimination scores. Hybrid S electrode users continue to improve on CNC scores over 1 to 2 years after activation. In the Hybrid S trial [14], 48% of the patients showed improvement in both SRT and CNC scores. Improvement on CNC testing ranged from 10% to 70% over preoperative scores for 73% of the patients with long-term followups [14]. For those implanted with the Hybrid L24, word recognition scores improved by 21% on average; one single patient had improvement from 5% to 95% on the Freiburg monosyllabic word test (FMT) [15].

Exceptional improvement in speech understanding is possible for patients in the combined mode with all electrodes activated: some patients score more than 90% on the CNC monosyllabic word test. Flex^EAS^ users had preoperatively open-set sentence recognition of 24% and after 12 months of use, scores averaged 71%. Monosyllable recognition also improved from 16% to 44% on average.

Improvements in discrimination tasks have also been observed in long-electrode users with preserved acoustic hearing. Patients who received the Med-El Combi40+ with 19 to 24 mm of insertion scored 75% on monosyllabic tests after 1 year, an important improvement from the 9% preoperative scores.

### 5.2. Hearing in Noisy Backgrounds

Distinguishing the correct words in a background of competing talkers is a challenging test for traditional cochlear implant users. Normal-hearing listeners have a signal-to-noise ratio (SNR) of −30 dB and −15 dB for competing talkers [35]. The average SNR of a long-electrode user is +3 dB for unmodulated background noise and +8 for multitalker babble (MTB), which means the talker has to speak 3 dB louder than the competing noise or 8 dB louder than MTB.

Hybrid S users do better than traditional cochlear implant patients, but not as well as normal-hearing listeners in noisy backgrounds. In a subgroup of 27 Hybrid S patients with at least 12 months of activation, SNRs ranged from −12 to +17 dB (average −9 dB) [14]. Patients who had a drop greater than 30 dB in their low-frequency hearing experienced a worse SNR.

Hybrid L electrode patients also improved their SNR preoperatively from 12.1 dB to 2.1 dB postoperatively [15]. Those with the Flex^EAS^ electrode changed their preoperative open-set sentence scores with a SNR of +10 dB from 14% to 60% after 1 year of activation [18].

Long-electrode users with preserved hearing in low frequencies also benefit when listening in noise. Med-El Combi40+ users with a low-frequency threshold of <80 dB scored better than patients with a cochlear implant and no residual hearing with a SNR of +5 dB [19]. Gstoettner et al. showed an increase in understanding scores from 13.1% preoperatively to 75% in the electric-acoustic stimulation condition [22].

### 5.3. EAS and Music Perception

It is known that traditional cochlear implant users have difficulties identifying and enjoying music because of the extremely complex encoded spectral information. They can usually distinguish lyrics to a certain degree but have significant trouble with pitch, timbre, and melody recognition [35, 36].

Music appreciation has been a part of the research protocol for the Hybrid S/L trials. Patients with preserved acoustic hearing have a distinct advantage over traditional cochlear implant users regarding pitch, lyrics, melody, and timbre of the instruments. When Hybrid users were provided with excerpts of easily recognizable American songs, they were able to correctly identify the songs 65%–100% of the time, similar to normal-hearing listeners. In tasks regarding pitch recognition and melody without lyrics, the Hybrid users still scored better than the traditional cochlear implant patients, but not as well as normal-hearing listeners did [36, 37].

Brockmeier et al. [38] studied music testing in EAS patients with long-electrode insertion (Med-El Combi40+). Thirteen EAS patients were compared with long-electrode users with no residual hearing and normal-hearing listeners. Subjects were matched by age and musical experience. EAS patients did as well as normal-hearing listeners on pitch discrimination and better than traditional cochlear implant users. However, EAS patients' scores in melody discrimination, instrument detection, and instrument identification were not significantly different from those of traditional cochlear implant users.

## 6. Future of Hybrid-Type Cochlear Implantation

Apical cochlear preservation may become a significant issue in the future. It is possible that treatments will emerge that require naïve cochlear tissue (i.e., hair cell generation or tissue transfer). This concern is relevant for families weighing the risks and benefits of bilateral cochlear implantation for deaf children. The Iowa CI research team has recently reported a series of 9 children with bilateral implants: one ear with a standard long electrode and the other with Hybrid S12 research electrode [39]. The children were only 12 to 24 months old at the time of implantation and they scored an average of 80 in Preschool Language Scale-3 (PLS-3), whereas the average score in the same test for children with bilateral standard electrodes is 83. A long-term followup is necessary to determine whether the trends of the preliminary data are long-lasting; a larger-scale study is being carried out.

## 7. Conclusion

Although hearing preservation in cochlear implantation is technically challenging, the importance of preserving residual low-frequency hearing cannot be underestimated in light of the added benefit of hearing in noisy atmospheres and music quality for EAS users. Electrode designs and processor technology are constantly being improved. Nowadays, patients undergoing hearing preservation surgery can expect a long-term low-frequency hearing preservation rate of 50–70%. Although surgeons have largely modified the initially described “soft surgery” technique over the years, there is not a unique protocol to be followed.

Few would have imagined the progress that has been made in the past 30 years regarding aiding deafened patients, and the future promises to be equally exciting. The potential EAS advantages highlight the value of the endeavors to preserve low-frequency hearing in the implanted ear and to continue amplification where appropriate. Further research is also needed to maximize outcomes for recipients with different degrees of hearing loss who use devices that combine electric and acoustic stimulation.
